# Covering all your bases: incorporating intron signal from RNA-seq data

**DOI:** 10.1093/nargab/lqaa073

**Published:** 2020-09-22

**Authors:** Stuart Lee, Albert Y Zhang, Shian Su, Ashley P Ng, Aliaksei Z Holik, Marie-Liesse Asselin-Labat, Matthew E Ritchie, Charity W Law

**Affiliations:** Epigenetics and Development Division, The Walter and Eliza Hall Institute of Medical Research, 1G Royal Parade, Parkville, Victoria 3052, Australia; Department of Econometrics and Business Statistics, Monash University, Clayton, Victoria 3800, Australia; Epigenetics and Development Division, The Walter and Eliza Hall Institute of Medical Research, 1G Royal Parade, Parkville, Victoria 3052, Australia; Epigenetics and Development Division, The Walter and Eliza Hall Institute of Medical Research, 1G Royal Parade, Parkville, Victoria 3052, Australia; Department of Medical Biology, The University of Melbourne, Parkville, Victoria 3010, Australia; Department of Medical Biology, The University of Melbourne, Parkville, Victoria 3010, Australia; Blood Cells and Blood Cancer Division, The Walter and Eliza Hall Institute of Medical Research, 1G Royal Parade, Parkville, Victoria 3052, Australia; Department of Medical Biology, The University of Melbourne, Parkville, Victoria 3010, Australia; Personalised Oncology Division, The Walter and Eliza Hall Institute of Medical Research, 1G Royal Parade, Parkville, Victoria 3052, Australia; Department of Medical Biology, The University of Melbourne, Parkville, Victoria 3010, Australia; Personalised Oncology Division, The Walter and Eliza Hall Institute of Medical Research, 1G Royal Parade, Parkville, Victoria 3052, Australia; Epigenetics and Development Division, The Walter and Eliza Hall Institute of Medical Research, 1G Royal Parade, Parkville, Victoria 3052, Australia; Department of Medical Biology, The University of Melbourne, Parkville, Victoria 3010, Australia; School of Mathematics and Statistics, The University of Melbourne, Parkville, Victoria 3010, Australia; Epigenetics and Development Division, The Walter and Eliza Hall Institute of Medical Research, 1G Royal Parade, Parkville, Victoria 3052, Australia; Department of Medical Biology, The University of Melbourne, Parkville, Victoria 3010, Australia

## Abstract

RNA-seq datasets can contain millions of intron reads per library that are typically removed from downstream analysis. Only reads overlapping annotated exons are considered to be informative since mature mRNA is assumed to be the major component sequenced, especially for poly(A) RNA libraries. In this study, we show that intron reads are informative, and through exploratory data analysis of read coverage that intron signal is representative of both pre-mRNAs and intron retention. We demonstrate how intron reads can be utilized in differential expression analysis using our *index* method where a unique set of differentially expressed genes can be detected using intron counts. In exploring read coverage, we also developed the *superintronic* software that quickly and robustly calculates user-defined summary statistics for exonic and intronic regions. Across multiple datasets, *superintronic* enabled us to identify several genes with distinctly retained introns that had similar coverage levels to that of neighbouring exons. The work and ideas presented in this paper is the first of its kind to consider multiple biological sources for intron reads through exploratory data analysis, minimizing bias in discovery and interpretation of results. Our findings open up possibilities for further methods development for intron reads and RNA-seq data in general.

## INTRODUCTION

Advances in gene profiling technology, such as RNA-sequencing (RNA-seq) have allowed researchers to study transcription in exquisite detail. Previously, quantitative gene expression analyses by microarray required prior knowledge of the sequences to be interrogated, limiting *de novo* discoveries and understanding of gene transcripts and alternative splicing especially at a high-throughput level. Most research efforts focused on gene-level information and comparison of genes that are differentially expressed (DE) between two or more groups. Whilst this is still the main focus for RNA-seq, the technology has the ability to examine sub-gene components such as at the transcript-level, exon-level, or even nucleotide base-level without prior sequence knowledge. As a result, there has been increased interest and effort into the study of transcript-level information, alternative gene splicing and gene intron retention (IR) at a global level using RNA-seq (1–3).

RNA-seq can be used to characterize and study many RNA types, including non-coding RNAs that regulate a diverse range of cellular processes (4,5), but the overwhelming majority of studies focus on messenger RNAs (mRNAs) which encode genes that are translated into protein. The most popular RNA selection protocol captures polyadenylated (poly(A)) RNA seeing that it is optimized for mRNA selection. In eukaryotes, poly(A) tails are synthesized to aid transportation of mature mRNA molecules from the nucleus to the cytoplasm, to increase molecule stability and for translation. Total RNA selection is also widely used, often including a step to deplete ribosomal RNA so that it does not compete with sequencing of mRNA. RNA expression values are highly correlated between the two RNA selection protocols, with a higher percentage of reads (≈3% more) mapping to protein coding genes in poly(A) RNA samples, and a higher percentage of reads (≈2.5% more) mapping to long non-coding RNAs in total RNA samples (6). The general assumption is that for protein coding genes the vast majority of RNA captured by the experiment are mature mRNA transcripts, as such, aligned sequencing reads are typically summarized only for annotated exons within genes. It is not common practice to quantify reads that overlap uniquely with intronic regions of a gene, perhaps because few reads are expected or due to suggestions that intron reads represent experimental and transcriptional noise (7) and/or are unusable in exon and gene quantification (8). However, intron reads can account for a significant proportion of sequencing reads (9).

In stark contrast, a small subset of studies have highlighted the use of intron reads, showing their correlation with measurements of nascent RNA (10) or by using the reads to study IR (3,11). The inclusion of intron reads into data analysis expands areas in which RNA-seq can be used to interrogate transcriptional biology at a high-throughput level, where for example IR has been shown to play important roles in inactivation of tumour suppressor genes (12) and during neutrophil (11) and erythroblast (13) differentiation. Whilst previous works on intron reads have been ground-breaking, they focus solely on one of the many aspects of transcription based on the biological interests of the study at hand. Gaidatzis *et al.* (10) thoroughly explored nascent transcription using intron reads in both poly(A) and total RNA libraries, but without mention of IR. Wong *et al.* (11) assumed that poly(A) RNA libraries contain only processed mRNAs, and successfully showed that genes with differentially retained introns are enriched in the cytoplasm. It is unclear, however, whether the same methods can or should be applied more generally to separate datasets. Perhaps, this would be determined by an expert with prior and thorough understanding of the underlying biology within a given dataset, but does not really allow an analyst to apply the methods to a randomly selected dataset to see if the results ‘make sense’. And yet, there is still little consensus on whether intron reads are informative to begin with.

In this paper, we summarize and explore the general characteristics of intron reads in a data driven manner. The work presented here allows for a novel perspective on technical, as well as multiple biological considerations when using intron reads. Demonstrating that intron reads are informative, we find that coverage profiles within intronic regions of poly(A) RNA libraries differ from that of total RNA libraries for genes that are relatively long. We observe that across most of the genes, their coverage patterns and strong correlation between exon and intron counts is consistent with our understanding of pre-mRNA signal. Amongst the pre-mRNA signal, for a human cell lines dataset we also select a small set of genes that have coverage profiles representative of IR. As a result of our exploratory work on intron reads, we have also made two novel methods available—*index* incorporates intron reads into differential gene expression (DGE) analyses; and *superintronic* is used to summarize read coverage for intronic and exonic regions. We expect that the results presented in this paper will better inform of how RNA-seq intron reads can be applied appropriately for various biological interests and further methods development.

## MATERIALS AND METHODS

### Datasets

*Human cell lines* of lung adenocarcinoma HCC827 and NCI-H1975 were cultured on three separate occasions by Holik *et al.* (14) giving three pseudo biological replicates. RNA was extracted from each pseudo biological replicate and split into two and prepared as poly(A) RNA and total RNA libraries. Raw sequencing reads were downloaded from the Gene Expression Omnibus (GEO) (15) under accession number GSE64098. Twelve libraries were examined for this dataset.

*Human immune cells* were sequenced by Linsley *et al.* (16) using a poly(A) RNA library preparation; GEO accession number GSE60424. RNA samples were taken of whole blood and six immune cell subsets, including pure populations of neutrophils, monocytes, B cells, CD4+ T cells, CD8+ T cells and natural killer (NK) cells. A total of 134 libraries were examined for this dataset.

*Mouse mammary cells* from female virgin mice with additional samples from mammosphere and the CommaD-βGeo (CommaD-bG) cell line were sequenced in a study by Sheridan *et al.* (17) to obtain poly(A) RNA libraries; GEO accession number GSE63310. Mammary cell populations include mammary stem cell-enriched basal cells, luminal progenitor-enriched (LP) and mature luminal-enriched (ML) cell populations. Nineteen libraries were examined for this dataset.

*Megakaryocytes and platelets* from mice were sequenced separately, with four and six libraries, respectively. Megakaryocytes were sequenced by Choi *et al.* (18) using a poly(A) RNA protocol; GEO accession number GSE116177. Poly(A) RNA libraries of platelets were sequenced by Chappaz *et al.* (19); GEO accession number GSE141161.

### Genomes and gene annotations

FASTQ files containing raw sequencing reads were aligned to the human *hg38* or mouse *mm10* genome using *subjunc* (20) with default parameters in the *Rsubread* software package (21). GENCODE’s main *Comprehensive gene annotation* file in GTF format was downloaded from https://www.gencodegenes.org for human (Release 27) and mouse (Release M12). Using ‘gene types’ (rather than ‘transcript types’) from Gencode, the annotation files were simplified by taking the union of two or more overlapping exons from transcripts of the same gene. The adjustment provides a simplification of genomic positions on each strand, such that each position is classified as belonging to ‘exon’, ‘intron’ or otherwise outside of an annotated gene. Three resultant annotation files were saved in standard annotation format (SAF)—exon annotation, intron annotation (region between exons) and genebody annotation (region spanning first to last exon). Our Supplementary Materials available at https://github.com/charitylaw/Intron-reads contain the scripts to process annotation files, together with other data analyses and supplementary figures.

### Intron and exon counts

Aligned reads were summarized by *featureCounts* (22) using exon annotation and genebody annotation separately to get gene-level *exon counts* and gene-level *genebody counts* respectively. Gene-level *intron counts* are calculated by subtracting exon counts from genebody counts.

Approximately 15% of genes had exon counts that were greater than genebody counts (by a median value of eight counts). This was due to our conservative approach of excluding reads that overlapped features in multiple genes during the read summarization step by *featureCounts* using the argument allowMultiOverlap=FALSE. Under this strategy, some reads were counted towards the exon count set but not the genebody count set. This happens when a read overlaps the exon in one gene and the intron of another gene—it is counted towards exon counts but not genebody counts due to its overlap of multiple genebodies but not multiple exons.

An alternate count strategy sets allowMultiOverlap=TRUE and does not result in higher exon counts than genebody counts. However, this gives ambiguous assignment of reads to counts via the multi-counting of reads, and can return a larger number of total counts than the original number of sequenced reads. This is not desired for our purpose of quantification and classification of reads into exon and intron sets.

Any gene with a larger exon count than genebody count, had its intron count adjusted to zero. Intron counts represent the gain in information when summarizing reads across the whole genebody relative to exonic regions only. Whilst there are other count strategies, such as counting exon–intron boundary reads separately or towards intron counts, we take this approach since our interest is in assessing whether the intron reads that are not typically used contain additional signal.

### Coverage patterns

Read coverage of intronic and exonic regions were calculated for poly(A) RNA and total RNA HCC827 human cell lines using our *superintronic* package, via the *Rsamtools* package (23). Genes of interest were restricted to protein coding genes on reference chromosomes, and we removed any genes that overlapped another to simplify the analysis and reduce coverage ambiguity. Genes were then further filtered if they were not expressed in the poly(A) RNA protocol (requiring at least three reads overlapping intronic and exonic regions). A total of 3262 genes were examined and categorized as short, regular or long (roughly 1087 genes in each category) by splitting the length of each gene into three bins by tertiles.

Using associated BAM files and GENCODE v27 annotation GTF, *superintronic* summarized the number of bases covered at a given coverage score for each gene and sample. For exonic regions and intronic regions, coverage scores were transformed to log_2_-scale using an offset of 0.5, and then normalized by dividing by each gene and each sample’s maximum log-coverage score. Normalized log-coverage scores, or *relative log-coverage* (relative to each gene’s maximum coverage), were divided into 20 windows along the length of each gene using the *GenomicRanges* package (24). To summarize coverage patterns across genes, *plyranges* (25) was applied to relative log-coverage scores by intersecting it with the positional windows. Each window’s mean coverage score (mean relative log-coverage) was calculated for each gene. Based on the position of the windows, they were further summarized across genes by taking its mean. The summary values were calculated separately for genes categorized as short, regular and long to represent general coverage trends along the gene body.

## RESULTS

### Intron reads are prevalent across datasets

**Figure 1.**
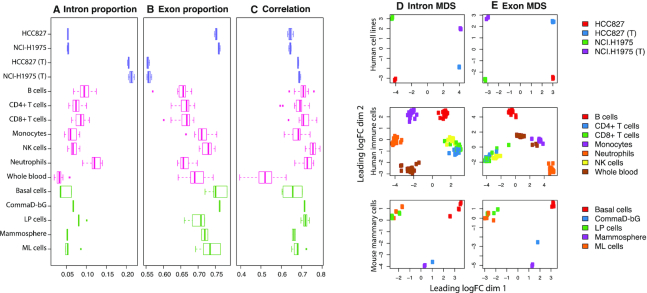
With libraries separated by biological and experimental groups, various statistics are summarized as boxplots across the datasets (distinguished by colour—purple for human cell lines, pink for human immune cells and green for mouse mammary cells) and total RNA samples labelled with a ‘(T)’. (**A**) Proportion of reads assigned to intron and (**B**) exon counts. (**C**) Pearson correlation of gene-level exon log_2_-counts (log-counts) and gene-level intron log-counts. Log-counts are calculated for genes expressed (count of three or more) in both intron and exon count sets, using an offset of 1. (**D**) MDS plots of log_2_-counts-per-million (log-CPM) values calculated using an offset of two for gene-level intron counts and (**E**) gene-level exon counts for each of the three datasets. MDS plots were created using *limma*’s (32) *plotMDS* function based on the top 500 most variable genes.

Taking a conservative approach, we quantify the number of intron reads that map entirely to an intron of a gene, excluding those that overlap an exon-intron boundary. Gene-level intron counts represent the extra counts one may obtain from within a gene when looking outside of annotated exons. The proportion of reads contributing to gene-level intron counts ranges from 2 to 14% with a mean value of 7% for poly(A) RNA libraries across three datasets examined (Figure 1A). A greater proportion of reads contribute towards gene-level exon counts, ranging from 57 to 78% with a mean of 69% (Figure 1B). Despite the relatively small proportion of intron reads, they amount to hundreds of thousands to millions of reads per library under typical sequencing protocols. For a library of size 30 million, the number of intron reads is ∼2.1 million (using the mean value of 7%).

A higher proportion of intron reads are found in total RNA libraries in comparison to poly(A) RNA libraries, as noted in prior studies (8,9). The mean proportion of reads contributing to intron counts and exon counts for total RNA libraries in human cell lines is 21 and 56%, respectively—a profound difference of roughly 15% more intron counts and 20% fewer exon counts when compared to corresponding poly(A) RNA samples. This equates to ∼6.3 million intron reads for a library of size 30 million. Intron and exon read proportions are fairly consistent for libraries within the same biological and experimental groups (such as within cell lines, cell types, tissues and RNA library protocol). Some variation in read proportions can be observed for different biological (e.g. neutrophils versus whole blood) and experimental (e.g. poly(A) RNA versus total RNA HCC827 cell line) groups. Within libraries, exon log-counts are positively correlated with intron log-counts (Figure 1C).

### Intron reads are informative and contain biological signal

In DGE analyses, plots of principle components analysis and multi-dimensional scaling (MDS) methods are commonly created from exon counts to provide an overview of the similarities and differences in transcriptional profiles in an unsupervised manner. To determine whether intron reads contain any biological signal, we applied MDS methods to intron counts instead. Samples cluster by experimental and biological groups in intron MDS plots across all datasets (Figure 1D) indicating that intron reads are informative, rather than a result of sequencing noise. As expected, samples also cluster by experimental and biological groups in exon MDS plots (Figure 1E).

Strikingly, the scales observed in the first and second dimensions of the intron MDS plots are comparable to that of the exon MDS plots even though there are roughly ten times fewer intron counts than exon counts. The distance between points on each plot give an indication of the typical log_2_-fold change (logFC) between samples in the top 500 most variable genes in each set of counts. In other words, the typical logFC between samples are similar for intron and exon counts.

Note that the first dimension of separation accounts for a larger proportion of variation in the data than the second dimension. The MDS plots for human cell lines indicate that intron read signal is significantly influenced by RNA selection protocols. The first dimension in the intron plot separates samples based on RNA selection method and accounts for 48% of the variation in the intron counts, whilst the second dimension relates to cell line identity and accounts for 18% of variation in the data. This is in contrast to the exon plot where the RNA selection protocol (second dimension) accounts for 26% of the variation in counts, and cell lines (first dimension) accounts for 37%. The type of RNA selection protocol used in library preparation has a greater influence on intron reads than exon reads.

### Comparing counts from poly(A) and total RNA libraries

The human cell line dataset allows us to further explore count differences between library preparation methods. Gene-level exon log-CPM values are similar between poly(A) RNA and total RNA libraries and have a very strong positive correlation (Supplementary Figure S1). Gene-level intron log-CPM values are also positively correlated but counts tend to be greater in total RNA than poly(A) RNA libraries (Supplementary Figure S1). Log-CPM values were calculated using an offset of two and by setting the library size as the sum of counts from exons and introns (log-RPKM values are calculated in the same way). This allows adjustment of intron and exon counts by the same sequencing depth per library, rather than an intron- or exon-specific proportion of the original sequencing depth.

Within libraries, we found that the majority ($56\%$ on average) of expressed, multi-exonic genes contain both intron and exon signal simultaneously. This was calculated by looking for the percentage of multi-exonic genes with counts of three or more in both intron and exon count sets, out of genes that are expressed. Expressed genes were defined as having a count of three or more in exon and/or intron counts. $32\%$ of expressed, multi-exonic genes were expressed in exon regions only (exon count ≥3, intron count ≤2) and $13\%$ of genes were expressed in intron regions only (exon count ≤2, intron count ≥3) on average.

To understand the nature of intron counts in relation to exon counts, we focus on the set of genes that are expressed in both regions, noting that total intron length of genes tend to become disproportionately large relative to total exon length (Supplementary Figure S2). Within poly(A) RNA libraries, intron and exon log-CPM and log-RPKM values are positively correlated (Supplementary Figure S3). Log-CPMs provide a reflection of the size of counts used as inputs to many analysis methods, and log-RPKMs are adjusted for length differences and provide a representation of read coverage levels. Intron coverage tends to be lower than exon coverage within the same gene, but the relative difference is quite stable across the genes (Supplementary Figure S3). The median difference between gene-wise exon log-RPKM and intron log-RPKM values is ≈5.1 across all poly(A) RNA HCC827 and NCI-H1975 cell line libraries, such that gene-wise exon coverage is roughly 34 times greater than intron coverage on average. Average intron coverage is affected by total length of intron regions in genes, such that genes with longer intron regions tend to have lower log-RPKM values (Supplementary Figure S3). Also, relative coverage of exons over introns increases as the total length of intron regions increases, and as exon log-RPKM increases (Supplementary Figure S3).

Similar trends are observed in total RNA samples (Supplementary Figure S3), though it is worth noting that for total RNA libraries the intron and exon counts have similar count size and dynamic range, and have stronger correlation of log-CPM and log-RPKM values between introns and exons. For total RNA libraries, exon coverage is roughly 10 times greater than intron coverage on average (median difference between gene-wise exon log-RPKM and intron log-RPKM values is ∼3.3 across all total RNA HCC827 and NCI-H1975 cell line libraries).

### Intron reads are predominantly from pre-mRNA

The notion that intron reads originate from pre-mRNA molecules rather than genes with retained introns is supported by the observation that gene-level exon counts tend to have a strong positive correlation with intron counts across all genes (Supplementary Figure S3), and that a large proportion of expressed, multi-exonic genes express intron and exon signal simultaneously. Assuming that IR is generally not widespread across all genes, a weak positive correlation is expected between intron and exon counts if intron reads were predominantly coming from genes with retained introns.

**Figure 2.**
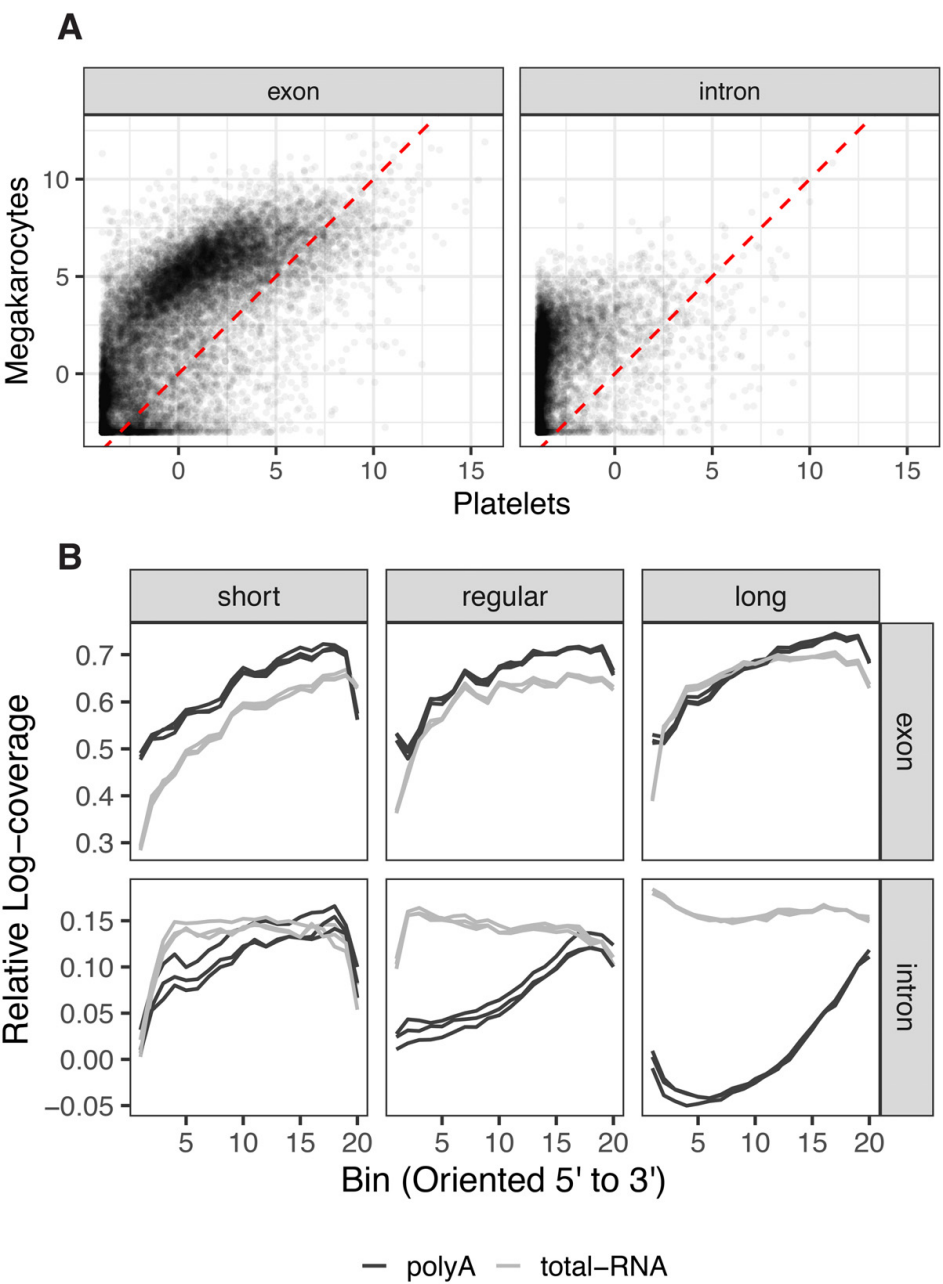
(**A**) Log-CPM values for exon counts in mouse megakaryocytes versus platelets; and for intron counts. Log-CPM values are calculated using the combined library size of intron and exon count sets, and the average value across biological replicates is plotted. Only multi-exonic genes and those that have a mean raw count of three or more in at least one count set are included. There are a total of four count sets—exon and intron counts for megakarytocytes, and exon and intron counts for platelets. (**B**) Coverage of intron and exon regions across the genebody. Top: Coverage patterns of exon regions across the genebody, separated into groups based on gene length. Coverage is represented by the mean of mean relative log-coverage, where relative log-coverage is calculated as local log-coverage divided by maximum log-coverage in a given gene. HCC827 cell line samples are depicted with black lines representing poly(A) RNA libraries R1, R2 and R3, and grey lines representing the corresponding total RNA libraries. Bottom: Coverage patterns of intron regions across the genebody.

To verify this, we examined intron and exon read counts from nucleated megakaryocytes and compared this to their anucleate platelet progeny. We observe overwhelmingly that intron reads are detected in megakaryocytes but are not detected in platelets (Figure 2A), leading us to conclude that the majority of intron reads correspond to pre-mRNA. Exon reads, on the other hand, are detected in both megakaryocytes and platelets.

Coverage patterns across the genebody of multi-exonic genes provide further evidence that intron signal is predominantly from pre-mRNAs with unspliced or partially spliced introns, where intron reads tend to be uniformly distributed in total RNA libraries and increasing gradually towards the 3′ end of genes in poly(A) RNA libraries (Figure 2B). Total gene length appears to play a part in intron coverage patterns. Genes under examination were catergorized by total gene length (length from first base in first exon to last base in last exon) such that a third were considered to be *short*, *regular* and *long* genes each. Coverage patterns were similar between poly(A) RNA and total RNA libraries for short genes, whilst patterns differed substantially for regular and long genes. Exon coverage patterns were similar for poly(A) RNA and total RNA libraries.

The coverage patterns were calculated by dividing per base log-coverage values in exonic regions by the maximum exonic log-coverage value in each gene. We refer to these values as *relative log-coverage* values in exonic regions (see ‘Materials and Methods’). The same is carried out for intronic regions. To summarize relative log-coverage over multiple genes, the values were averaged within windows in each gene before taking the average of windows across genes.

**Figure 3.**
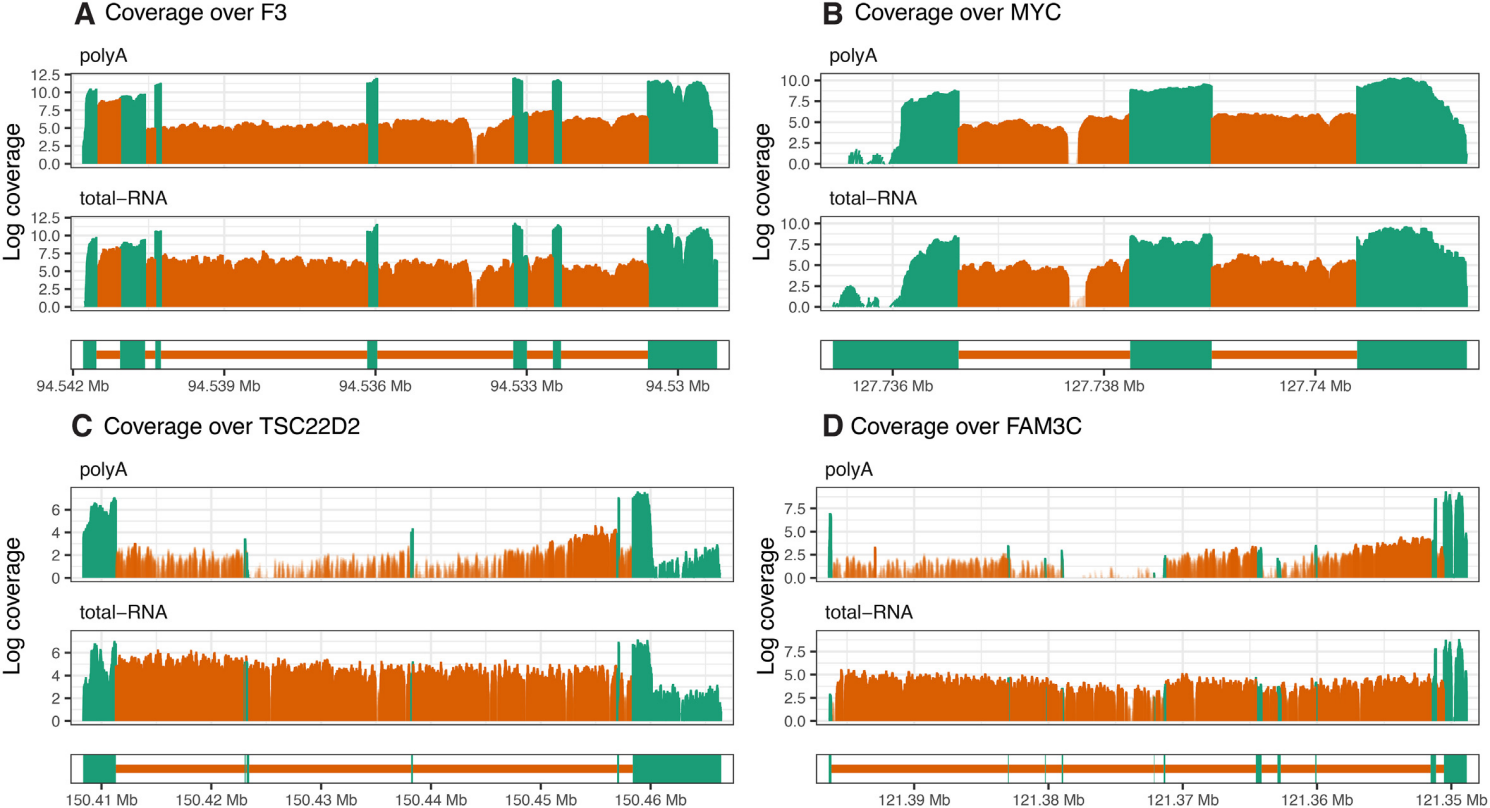
Log-coverage in exon regions (green) and intron regions (orange) in HCC827 poly(A) RNA R1 library (top) and HCC827 total RNA R1 library (bottom) are displayed for two short genes, (**A**) F3 and (**B**) MYC, and two long genes, (**C**) TSC22D2 and (**D**) FAM3C. Genes are oriented from 5′ to 3′, from left to right. These genes were selected based on having a high median intron log-coverage. Intron regions with high expression (log-coverage greater than three) are highlighted by a shade of darker orange.

Confirming the same patterns for individual genes, coverage profiles of two short genes F3 and MYC are observed to be similar between RNA library preparation (Figure 3A and B). In contrast, the coverage profiles of two long genes TSC22D2 and FAM3C differ at the 5′ end where poly(A) RNA libraries are observed to have deflated intron coverage relative to the 3′ end, as well as relative to total RNA libraries (Figure 3C and D). Reduction in read coverage at 5′ introns for poly(A) RNA libraries explain why poly(A) libraries are observed to have relatively low proportions of intron reads (Figure 1A) and dynamic range in log-CPM values relative to total RNA libraries (Supplementary Figure S3).

Relative gene-level contribution by pre-mRNAs is low compared to mature mRNAs for the majority of genes, as reflected in low intron log-RPKM relative to exon log-RPKM values (Supplementary Figure S3). If intron and exon log-RPKM values provide an estimate of the relative proportions of pre-mRNA and mRNA molecules captured, then on average roughly 1 pre-mRNA molecule is captured for every 10 molecules in a sequencing experiment (since exon coverage is roughly 10 times greater than intron coverage in total RNA libraries, and total RNA libraries have uniform intron coverage). Unless the sequencing experiment is carried at very high depths, intron signal may not be detected for genes with relatively short intron regions since pre-mRNA levels are low, whilst genes with long intron regions have greater ability to accumulate sequencing reads over the gene.

### DGE analyses of transcriptional activity using intron and exon counts

Classical DGE analyses are performed on gene-level exon counts, where in light of results from the previous section we have an understanding that the associated reads originate from mRNA as well as pre-mRNA molecules. Previously, signal from microarrays designed with exonic and intronic probe sets were used to study transcriptional dynamics of pre-mRNAs and mRNAs (26). For RNA-seq data, we propose a method that complements the classical DGE analysis and includes intron counts to measure changes in early transcriptional activity. We call our method *index*, **in**tron **d**ifferences to **ex**on, a DGE method categorizing genes by significance and directional changes in intron and exon counts. Relative to a classical DGE analysis which requires gene-level exon counts and some information about the experimental design and comparisons of interest, *index* simply requires an addition of gene-level intron counts. *Index* is an R package which is available to download and install at https://github.com/Shians/index.

**Figure 4.**
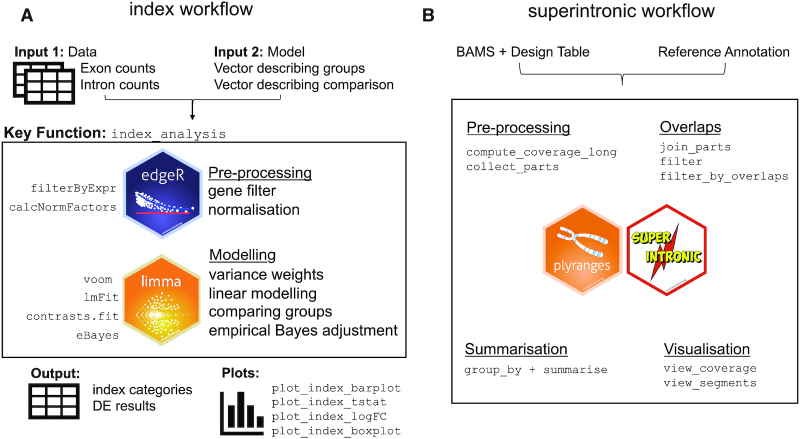
Overview of the *index* and *superintronic* workflows. (**A**) Beginning with matrices containing exon and intron counts by gene and vectors describing an experimental design and contrasts of interest, *index* uses *edgeR* for normalization and gene filtering. Then using *voom*, DE results are computed and are categorized according to significance in exon and intron counts. (**B**) The modular workflow for exploratory genomics data analysis using *superintronic* and *plyranges*. The software requires BAM files, gene annotation and information on experimental design as input prior to any computation. Each stage can be performed independently depending on the end goal of the analysis. The pre-processing steps compute coverage as a long form *GRanges* object in parallel and with respect to an experimental design. The annotation can also be used to construct the exonic and intronic parts of a gene. Region-based overlaps or filters can be performed to either zoom in on a gene of interest or to split coverage over intron and exon parts. Coverage can then summarized over regions with respect to a design or a sample using a statistical summary such as the mean, sum or standard deviation or any suitable R function. A suite of visualization functions are provided to look at coverage in the context of gene annotations or for finding interesting regions of coverage.

The *index* workflow (Figure 4A) is carried out on genes that are expressed in both intron and exon regions. Firstly, sufficiently large intron and exon counts are selected by the *filterByExpr* function in *edgeR* (27,28). Trimmed mean of M-values (TMM) normalization (29) is then carried out on intron and exon count sets separately using the combined library size for samples (sum of both pre-filtered intron and exon counts). This is a variation on the standard library size calculation which only sums counts within a single count set. Intron and exon counts evaluated based on standard library sizes will be affected by intron and exon read proportions (Figure 1A and B) which vary between samples, groups and experiments and gives a poor estimate of original sequencing depth. The combined library size is used also for downstream calculations, such as in obtaining log-CPM values by *voom* (30).

DE genes are obtained for intron and exon counts separately following a standard *limma-voom* pipeline (31) where log-CPM values and variables of interest are modelled on a Normal distribution with precision weights calculated by *voom*. Moderated *t*-statistics are calculated for each gene using Empirical Bayes’ methods (32,33) and *P*-values are adjusted for multiple testing by controlling the FDR (34). Genes with an adjusted *P*-value of less than a nominal cutoff are considered to be DE.

*Index* categories are formed based on significance in intron and exon counts: **+** for genes upregulated in both intron and exon counts, **-** for downregulated in intron and exon counts, **exon+** and **exon-** for up- and downregulated in exon counts only, **intron+** and **intron-** for up- and downregulated in intron counts only, **mixed+-** for upregulated in exons and downregulated in intron counts and **mixed-+** when in the opposite direction and **0** for no significant difference in either exon or intron counts.

The *index* software performs analysis on intron and exon DGEList objects (a native object of *edgeR*) to classify genes into the respective *index* categories. *Index* outputs categories assigned to each gene, *limma*-style tables of DGE results for introns and exons, and other data used by the software to create plots. This allows the *index* analysis to be easily performed on any dataset where intron and exon counts can be obtained separately.

### *Index* analysis of human cell lines and immune cells

**Figure 5.**
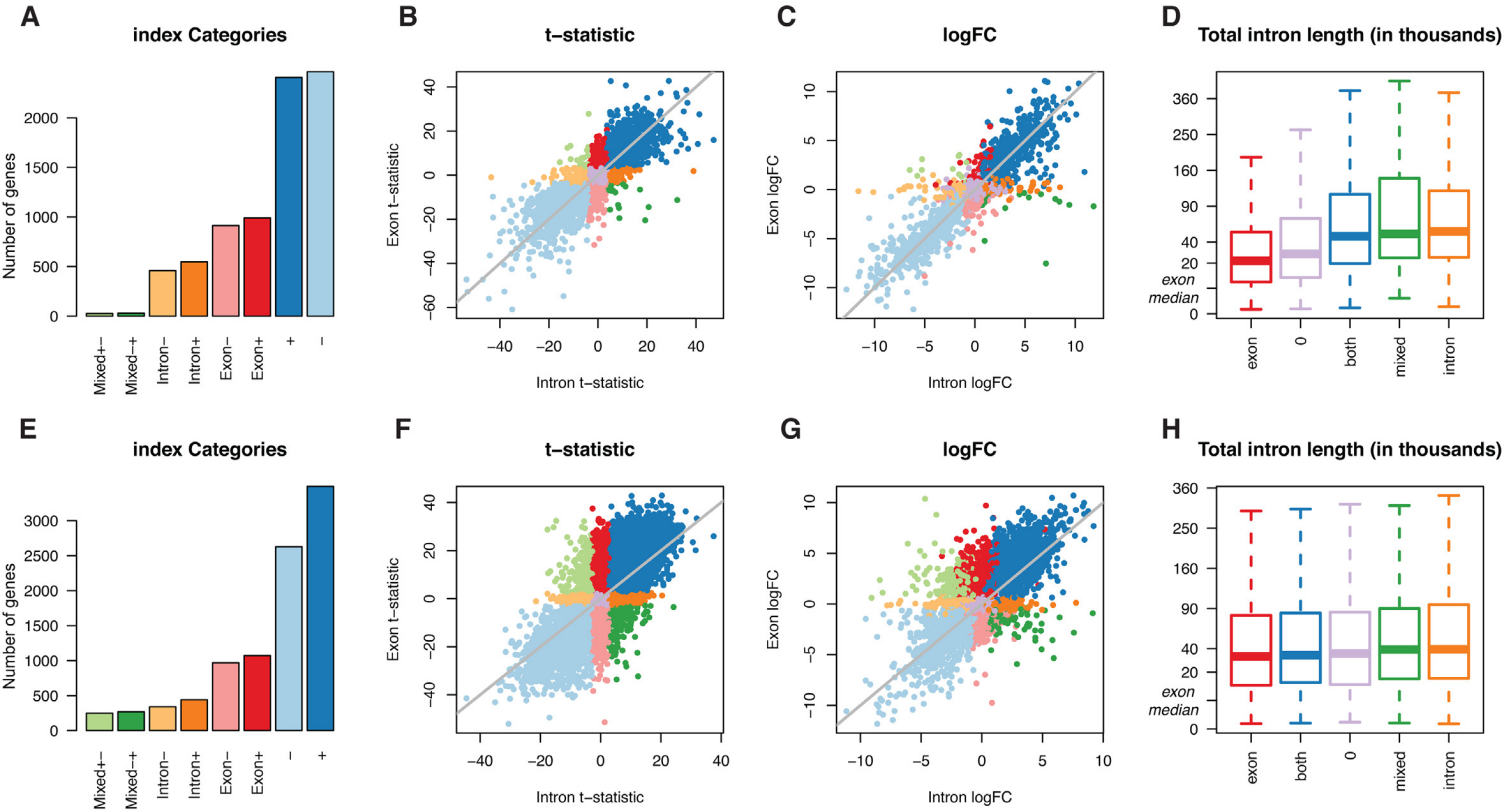
Results for DGE analysis of intron and exon counts using *index*. (**A**) Number of significant genes in *index* categories for NCI-H1975 versus HCC827 cell lines in total RNA libraries, with (**B**) t-statistics from exon counts plotted against those from intron counts and (**C**) logFC values from exon counts plotted against those from intron counts, where colours are associated with different *index* categories. (**D**) Distribution of total intron length ordered by median total intron length of *index* categories. The categories are combined here, such that genes that are upregulated or downregulated for both intron and exon counts are reassigned to *both*; *exon* for genes up- or downregulated for exon counts only, similarly for *intron* and *mixed*. A square-root scale is used along the vertical axis, with the median total exon length (5126 bases) marked as a reference. (**E–H**) Similar plots are displayed for a comparison of monocytes and neutrophils in the immune cells dataset.

An *index* analysis comparing NCI-H1975 versus HCC827 cell lines in total RNA libraries reveals that the majority of genes are DE in the same direction between intron and exon counts—2406 genes upregulated (+) in NCI-H1975 and 2464 downregulated (−) using an adjusted *P*-value cutoff of 0.01 (Figure 5A–C). Genes DE by exon counts only form the second biggest group, with 989 genes upregulated (exon+) and 914 genes downregulated (exon-) in exon counts. Interestingly, these genes tend to have short intron regions (Figure 5D). There are 547 genes upregulated (intron+) and 459 genes downregulated for intron counts only (intron-), where genes tend to have relatively long intron regions. Similarly, genes DE in opposite directions also have relatively long intron regions—a small group of 25 genes upregulated in exon counts but downregulated in intron counts, and 29 genes upregulated in intron counts but downregulated in exon counts. The analysis was carried out on 11608 genes after lowly expressed genes were filtered out.

An identical analysis comparing monocytes versus neutrophils in the immune cells dataset similarly reveals that the majority of genes are DE in the same direction between intron and exon counts—3491 genes are upregulated (+) in neutrophils relative to monocytes and 2628 downregulated (−) using an adjusted *P*-value cutoff of 0.01 (Figure 5E–G). Again, genes DE by exon counts only form the second biggest group, with 1073 genes upregulated (exon+) and 969 genes downregulated (exon−) for neutrophils in exon counts. These genes again tend to have shorter intron regions, but the difference in length between *index* catergories is subtle compared to that of cell lines (Figure 5H). There are 441 genes upregulated (intron+) and 340 genes downregulated (intron-) for intron counts only, where genes again tend to have longer intron regions. The number of genes DE in opposite directions between intron and exon counts is larger for this comparison than in cell lines, with 249 genes upregulated in exon counts but downregulated in intron counts (mixed+−) and 269 genes downregulated in exon counts but upregulated in intron counts (mixed−+). The analysis was performed on 10789 genes after lowly expressed genes were filtered out.

### *Index* analysis detects additional DE genes

The *index* DGE analyses demonstrate that transcriptional changes detected by exon counts are similar to those detected by intron counts. This is expected since exon counts represent mRNA and pre-mRNA levels, whilst intron counts largely represent pre-mRNA levels. For most genes, similarity between intron and exon logFCs (Figure 5C and G) indicate that pre-mRNA and mRNA levels are simultaneously up- or downregulated at similar proportions between groups.

We hypothesize that genes categorized as intron+ or intron− mostly contain changes in pre-mRNA levels only. To verify this, we examine read coverage profiles for genes with the largest expression differences in cell line samples (largest absolute *t*-statistics in intron+ and intron− categories). We observe that genes in the intron+ and intron− categories have coverage profiles that are consistent with what we would expect of changes in pre-mRNA levels, such that reads are covering most of or all of the genebody for one cell line and higher than that of the other cell line (see ‘Analyses’ page in Supplementary Materials).

Assignment of genes into different *index* categories is associated with total intron length of a gene (Figure 5D and H), such that genes DE for exon counts only tend to have relatively short intron regions. Naturally, these genes are unlikely to accumulate high intron counts due to low coverage and short region lengths, thus lacking power during statistical testing. On the other hand, genes DE for intron counts only tend to have relatively long intron regions; due to their length they are able to accumulate high intron counts even if coverage levels are low, giving them a power advantage when testing for differential expression.

In other words, exon+ and exon− genes may also contain changes in intron regions even though they remain undetected. Alternative explanations for observing significant changes in exon counts only are less likely, for example, that there are no pre-mRNAs observed, or that pre-mRNA levels are consistent between groups. The former is contradicted by Supplementary Figure S3 which shows high intron coverage for genes with short intron regions, and the latter is unlikely to be a trait specific to genes with short intron regions. Similarly, intron+ and intron− genes may also contain changes in exon regions even though they remain undetected.

If intron counts represent pre-mRNA levels, then any change observed between groups in intron counts should also be reflected in exon counts. However, exons are unlikely to accumulate high counts over its relatively short regions if pre-mRNA (and mRNA) levels are very low. If genes have retained introns or differentially retained introns in one group versus another, it is also possible for genes to be detected as DE in intron counts. Intron+ and intron− genes can be compared against a list of genes detected with retained introns (see *superintronic* in next section). Given that significance is influenced by total intron length, it is possible that exon+, exon−, intron+ and intron− genes may be reclassified into + and − *index* categories if sequencing was performed at greater depths.

Mixed+− and mixed−+ genes form a relatively small set of genes relative to other *index* categories. Biologically, a simultaneous increase in pre-mRNA levels and decrease in mRNA levels between two groups can induce changes in opposing directions between intron and exon counts. For example, this may occur during nonsense mediated decay (35), where post-transcriptional regulation may lead to a decrease in mature transcripts.

An *index* DGE analysis adds an extra layer of information by overlapping intron and exon results, where additional DE genes are detected that are not observed in a classic DGE analysis alone. The *index* method has increased power in genes with long intron regions, where high counts can be detected for low coverage genes. A classic DGE analysis, by *limma-voom* or like methods, followed by an *index* DGE analysis allows researchers to make use of a larger proportion of reads that are already sequenced and available to them to detect additional DE genes.

### *Superintronic*: an exploratory approach to detecting genes with IR

Considering IR also as another possible source of intron reads we propose a new method using our *superintronic* software to explore intron signal directly from aligned sequencing data with the assumption that most intron reads do not point to IR but pre-mRNA instead. *Superintronic* is an R package that is available to download and install at https://github.com/sa-lee/superintronic. It extends the *plyranges* Bioconductor package (25) for genomics data analysis to develop a simple and modular interface for performing exploratory genomics data analysis via coverage estimation. Each aspect of the *superintronic* data analysis workflow as it has been applied for exploring intron signal is outlined in Figure 4B.

Our software records the per base coverage over intron and exon regions of each gene, with the option of storing these per sample or summarized over variables in the experimental design such as by biological group or by RNA library preparation. Coverage scores are normalized using a log_2_-transformation with an offset of 0.5 to get log-coverage values for which intron and exon summary statistics are constructed for each gene (described below). Within *superintronic*, a suite of visualization tools to construct coverage plots for genes with intron and exon structures and scatter plots are provided.

### *Superintronic* finds genes with IR-like coverage profiles in human cell lines

**Figure 6.**
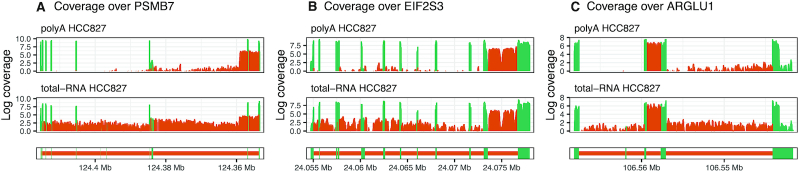
*Superintronic* selects genes with IR-like coverage profiles in poly(A) RNA HCC827 cell lines. Genes (**A**) PSMB7, (**B**) EIF2S3 and (**C**) ARGLU1 are highlighted out of 43 genes selected. Whilst the analysis was carried out on poly(A) RNA libraries, coverage is shown for both poly(A) RNA HCC827 (top) and total RNA HCC827 (bottom) samples to ensure that results are not an artifact of RNA library preparation. Coverage is oriented from 5′ to 3′, with exon regions coloured green and intron regions coloured orange.

Using *superintronic*, poly(A) RNA HCC827 cell lines were examined for genes with IR after selecting genes in the hg38 reference that were protein coding, did not overlap any other gene and were placed on the main contigs—a total of 6606 genes. These genes were then split into intron and exon regions and intersected with the coverage of each sample. Per gene intron and exon summary statistics, mean and standard deviation, were computed on log-coverage values. We selected genes enriched for IR-like coverage profiles by looking for ‘expressed’ genes, where for a substantial number of intron bases its coverage is much higher than other intron features within the same gene whilst having similar expression levels to the exon features. To do this we used the following thresholds—genes had an average exon log-coverage of greater than two (corresponding to the mean of average exon log-coverage values across all genes), a standard deviation of intron log-coverage >1.5 (corresponding to the mean of intron standard deviation values across all genes), and genes with a large number of intron bases with log-coverage greater than two (top 1% of genes). The thresholds were chosen after examining distributions of the summary statistics (Supplementary Figure S4). Forty-three genes met these criteria, where a manual check of coverage profiles revealed that 36 genes indeed appear IR-like (see ‘Analyses’ page in Supplementary Materials). We highlight three of these genes in Figure 6. The coverage of remaining seven genes appear to be more pre-mRNA-like, with large variation in intron coverage, where at its peak it is expressed similarly to exon features.

We use poly(A) RNA samples in our analysis for consistency with previous studies on IR. In theory, total RNA samples may be more appropriate for this exercise since it is less biased towards the 3′ end, where high 3′ intron coverage as a result of 3′ bias in poly(A) RNA libraries can be mistaken as a 3′ retained intron. For this reason, we review both the coverage of poly(A) RNA and total RNA samples in our Supplementary Materials for all selected genes to ensure that retained introns found in poly(A) RNA samples are not an artifact of RNA library preparation.

If differentially retained introns are of interest, one could simply run *superintronic* on two conditions separately and compare lists of IR-like genes between groups. We found that differences between the lists of IR-like genes were concordant with *index* results for genes DE by intron counts, as expected (see ‘Analyses’ page in Supplementary Materials). For example, 14 genes were uniquely selected as IR-like in total RNA HCC827 cell line when compared to total RNA NCI-H1975 cell line using *superintronic*, where 13 of those genes were also found to be DE in intron counts by *index*, and in the expected direction. Similarly, 19 genes were uniquely detected as IR-like in total RNA NCI-H1975 by *superintronic*, 16 of which were also detected as DE in intron counts by *index*. Additionally, 12 genes which were found to be IR-like in both cell lines appear to have varying intron expression according to *index* since it is found to be DE in intron counts.

Whilst genes that are uniquely IR-like were concordant with directional changes in intron counts, we did not find that IR-like genes played a large part in *index* gene categorization. This was mostly expected since thousands of genes were detected as significantly DE by *index* and a relatively small number of genes were detected as IR-like by *superintronic*. Specifically, IR-like genes did not overlap with one particular *index* category (see ‘Analyses’ page in Supplementary Materials). Of genes that are classified as intron+ or intron−, only 3 out of 1006 genes were also detected by *superintronic* as IR-like. In the ‘mixed’ category, 2 out of 54 genes were also detected as IR-like.

## DISCUSSION

The work presented in this paper provides a broad view of the characteristics and expression patterns associated with intron reads in model organisms, namely human and mice. Incomplete gene annotations may result in misclassification of reads as intron reads. We looked into this by examining split reads as indicative of splice events, and found the effect of unannotated exons in our analyses of human cell line data to be minimal. For example, HCC827 cell line samples have a median of zero split reads within introns (third-quartile of 0.33), as compared to a median number of 38 split reads in exons (third-quartile of 121.33). This demonstrates that splice events are rare within annotated intronic regions, and suggests that unannotated exons play a very minor role in the results we presented overall. For example, only 2% of genes in any of the *index* categories contain introns with 10 or more split reads (see ‘Analyses’ page in Supplementary Materials).

Interestingly, 41% of *superintronic*’s IR-like genes contain introns with 10 or more split reads. However, we do not believe that this indicates that the genes were detected because of unannotated exons since our thresholds ensure the selection of high coverage regions that are much larger than the typical exon. For the understanding of complex splice events, further biological validation of the IR-like genes is of interest but beyond the scope of this paper.

For organisms that are poorly annotated, the number of reads misclassified as intron reads may be significantly inflated. In such cases, ‘intron reads’ that are split reads can be useful for identifying new exons. DGE analyses using genebody counts or by our *index* method would naturally incorporate the transcriptional changes within unannotated exons of annotated genes.

Using gold standard differential expression methods, *index* selects and categorizes genes of interest based on *P*-values from moderated *t*-statistics that are adjusted for multiple testing and the direction of change. This is statistically more sophisticated than the *EISA* method which uses intron and exon logFCs alone and provides no prioritization of genes of interest—this is, however, sufficient for their purpose of categorizing genes as sets, and determining whether the biological system under study is driven transcriptionally or post-transcriptionally as a whole. In contrast, *index* looks for DE genes using evidence from intron and exon counts. *Index*’s logFC plot (Figure 5C and G) is analogous to *EISA*’s main result and logFC plot.

The presence of pre-mRNA in poly(A) RNA libraries may be somewhat surprising since the RNA library preparation is optimized for mRNA selection. However, pre-mRNA can be captured by both poly(A) RNA and total RNA protocols since transcription from DNA to a primary RNA transcript, 3′ cleavage of the RNA molecule and polyadenylation can be completed before splicing is complete at the 3′ end because the splicing mechanism requires a relatively long processing time (36) – this is regardless of whether genes are co-transcriptionally (9,37–38) or post-transcriptionally spliced. Evidence supporting this includes the presence of poly(A)-positive molecules in the nucleus that are larger than final mRNAs in the cytoplasm (36).

Handling of RNA in preparation for sequencing results in a degree of fragmentation of the original molecule regardless of the level of care taken during this process. This has minimal downstream effects on total RNA libraries since 3′ and 5′ fragments are selected randomly. However, the selection of poly(A)-positive RNA molecules in poly(A) RNA libraries bias fragments at the 3′ end whilst 5′ fragments are lost in the process. This results in 3′ coverage bias in poly(A) RNA libraries (Figure 2B)—demonstrated also by Lahens *et al.* (39) in their study on technical biases introduced during generation of sequencing libraries. Shorter genes with fewer and/or shorter introns are less affected by fragmentation than genes with long RNA molecules, thus coverage profiles are more similar between total RNA and poly(A) RNA libraries for these genes (Figure 2B). Read coverage in total RNA libraries may provide a more accurate representation of the original RNA molecule than in poly(A) RNA libraries, especially in long genes. In poly(A) RNA libraries, the inflated 3′ exon expression and 3′ ‘background’ intron signal may negatively impact on methods for *de novo* transcriptome assembly and transcript quantification. total RNA libraries which have uniform ‘background’ intron signal is easier to model in theory and should be better suited to such applications. Moreover, existing IR detection methods applied to poly(A) RNA sequencing libraries will have increased difficulty in interrogating introns residing towards the 5′ end of genes.

We have explored intron signal arising in this context via coverage estimation performed using *superintronic* and found that we can visualize IR-like coverage profiles by using simple summary statistics generated from genomic overlaps. Our method differs from existing methods such as *IRFinder* (40) and *IsoformSwitchAnalyzeR* (41) in that it can detect IR-like coverage profiles in individual conditions, rather than differences between two conditions. Naturally, the detection of differentially retained introns within a gene should firstly include a retained intron in at least one condition, and secondly contain differences in the expression of the intron. Since detection of either of these steps are non-trivial, we believe that it is more important to focus on detecting retained introns directly via visualization of ‘interesting’ coverage profiles. However, a downside of our approach is that does not perform any statistical inference on a given coverage profile to say whether a region is truly IR-like. In this way we see the use of *superintronic* as both complimentary to *index* and useful in its own right for flexibly summarizing reads with respect to an experimental design, it can be used to perform quality control on *index* results interrogate exon/intron count data further by viewing coverage profiles. We have also found that estimation of coverage profiles can provide a visual check of differential IR results from other methods; for example we have observed significant results in poly(A) RNA libraries tending towards the 3′ end of genes for both *IRFinder* and *IsoformSwitchAnalyzeR* (Supplementary Figure S5). Although the results are not directly comparable, we note that 23% of genes uniquely detected as IR-like in either of the cell lines using *superintronic* overlap with differential IR results using *IRFinder*’s generalized linear models method. Whilst this is not a big overlap, it shows some level of consistency between the methods. On the other hand, none of the uniquely IR-like genes are in common with results from *IsoformSwitchAnalyzeR* (see ‘Analyses’ page in Supplementary Materials).

Compared to bulk RNA-seq, single-cell RNA-seq (scRNA-seq) data have much smaller library sizes and relatively high proportions of intron reads leading to much interest in the incorporation of intron reads in scRNA-seq data analyses (42). Though yet to be tested, DGE analysis by *index* should theoretically perform well on scRNA-seq data since it increases the number of testable genes and libraries by increasing the amount of information used.

The work presented in this paper explores multiple origins of intron reads and signal in RNA-seq data. We demonstrate the usefulness of applying intron reads to study multiple aspects of transcriptional biology, and provide tools to interrogate changes in pre-mRNA and mRNA levels, as well as genes with IR-like coverage profiles. Biological validation of our results was not carried out and is beyond the scope of this paper, since the intention was to make conclusions using a data-driven approach. However, further work includes closer examination into pre-mRNA-specific and IR-specific signal, such as by using full length transcripts by long-read sequencing by Pacific Biosciences (43) or Oxford Nanopore Technologies (44). It is also of interest to examine intron reads in datasets with RNA from cytoplasmic and nuclear fractions versus whole cell.

## CONCLUSION

Intron reads are prevalent at small to moderate proportions in RNA-seq datasets, however, they provide signal that can distinguish between biological and experimental groups. Harvesting these extra reads as pre-mRNA signal, DGE analysis can be carried out more thoroughly with the addition of intron counts into *index*. The extra layer of analysis enables distinction between changes in pre-mRNA and mRNA signal enhancing the understanding of transcriptional changes and dynamics under study. IR remains an important mechanism in biology and can be explored through the use of *superintronic*, which can discover genes with IR-like coverage profiles.

## Supplementary Material

lqaa073_Supplemental_Files
