# Supplementary material for: Covering all your bases: incorporating intron signal from RNA-seq data
Source: NAR Genom Bioinform. 2020 Sep 22;2(3):lqaa073. doi: 10.1093/nargab/lqaa073 (PMC7671406; doi:10.1093/nargab/lqaa073)
Supplement: lqaa073_Supplemental_Files [file lqaa073_supplemental_files.zip › Sup Fig Captions.docx]

**Sup Fig 1 - Between library comparisons.pdf** : Comparing RNA protocols for gene-level exon log-CPM (left) and gene-level intron log-CPM values (right) for human cell line HCC827 R1, R2, R3, human cell line NCI-H11975 R1, R2, and R3 (in order of plots shown).

**Sup Fig 2 - Intron length.pdf** On a log2-scale, the relative length of introns compared to exons are plotted against the length of introns. The exon length of a gene is calculated as the sum of all exon regions as defined in the exon annotation; similarly for intron length. Log-values are calculated using an offset of 0.001.

**Sup Fig 3 - Within library comparisons.pdf** : From left to right, intron versus exon log-CPM, intron versus exon log-RPKM, intron log-RPKM versus total intron length, relative coverage between exon and intron regions versus total intron length, and relative coverage versus exon log-RPKM; with poly(A) RNA (top row) and Total RNA libraries (bottom row). The plots are displayed for human cell line HCC827 R1, R2, R3, human cell line NCI-H11975 R1, R2, and R3 (in order of plots shown).

**Sup Fig 4 - Superintronic summary values.png**: Scatterplot matrix of summary values from running superintronic on the poly(A) RNA HCC827 cell line, with associated density plots for exon mean (exon_mn), intron mean (intron_mn), intron standard deviation (intron_sd) and number of intron bases above the threshold. More details can be found in the [superintronic vignette](http://htmlpreview.github.io/?https://github.com/sa-lee/analysis-superintronic/blob/master/Rmd/01-superintronic.html)

**Sup Fig 5 - Example DIR genes from IRF and ISA.pdf** Example genes with differentially retained introns between poly(A) RNA human cell lines as detected by IRFinder and IsoformSwitchAnalyzeR, visualised as coverage plots facetted by cellline (a) HNRNPL detected by IRFinder GLM test (b) NBEAL2 detected by IRFinder Audic and Claverie test and (c) HLA-B detected by IsoformSwitchAnalyzeR DEXSeq test. Coverage is orientated from 5' to 3', with exon regions colored green and intron regions colored orange.
