# Supplementary figures and images for: Covering all your bases: incorporating intron signal from RNA-seq data
Source: NAR Genom Bioinform. 2020 Sep 22;2(3):lqaa073. doi: 10.1093/nargab/lqaa073 (PMC7671406; doi:10.1093/nargab/lqaa073)

Supplementary Figure S1

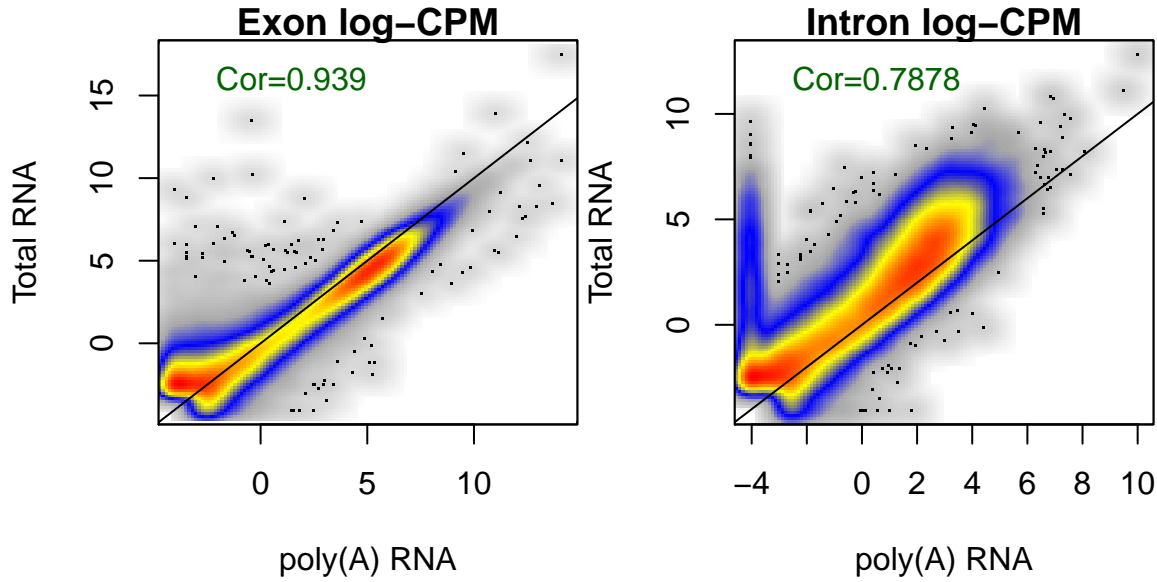

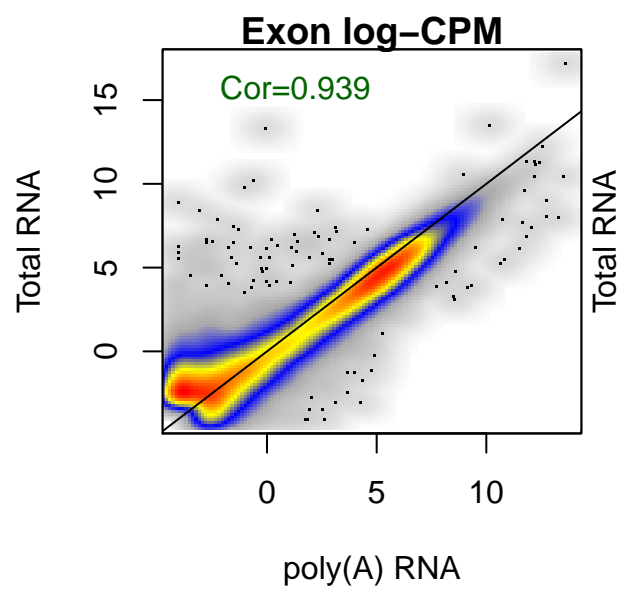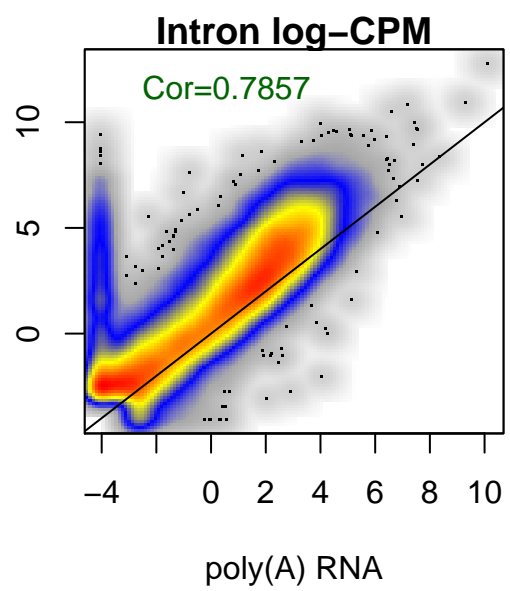

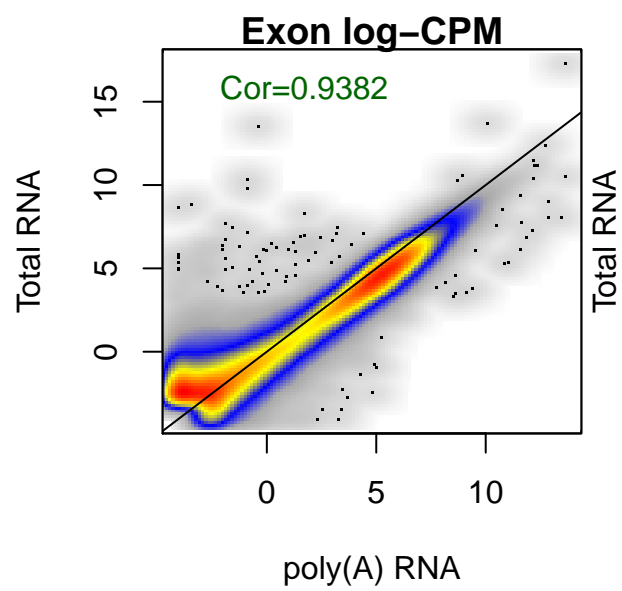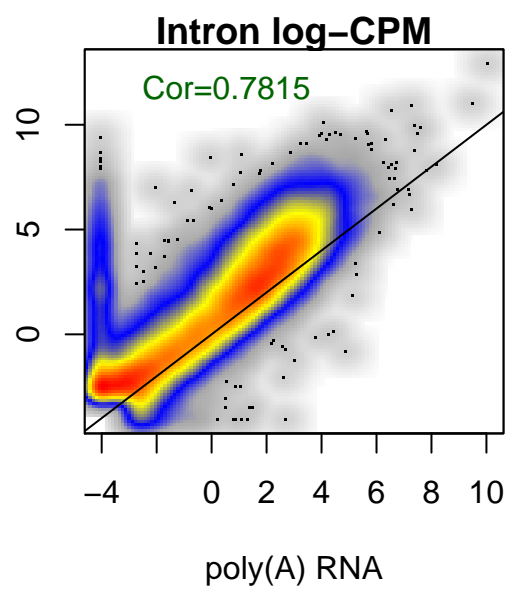

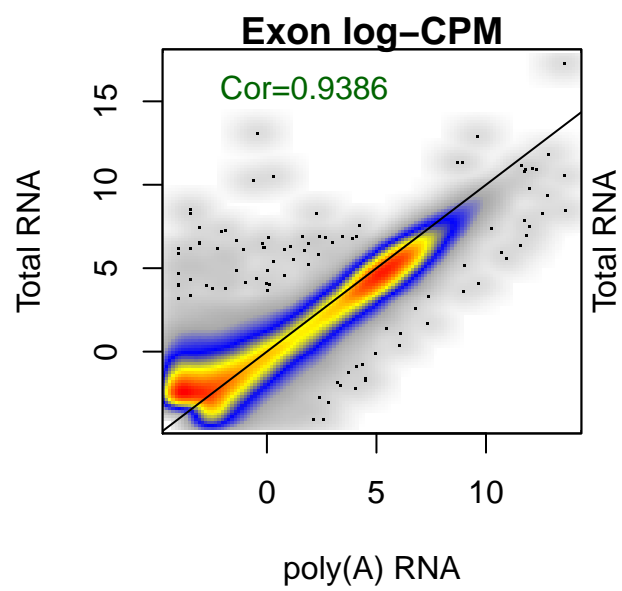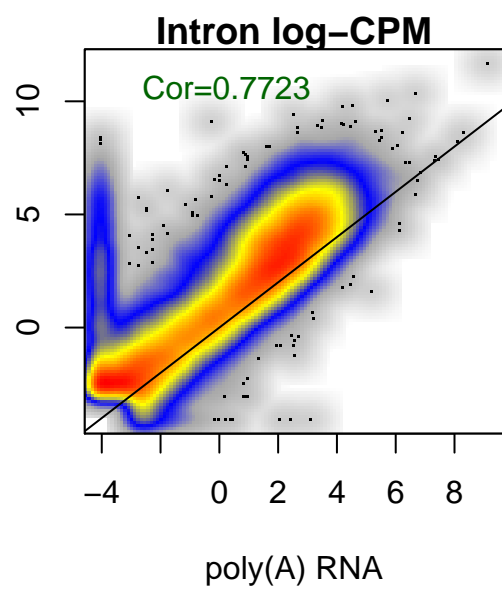

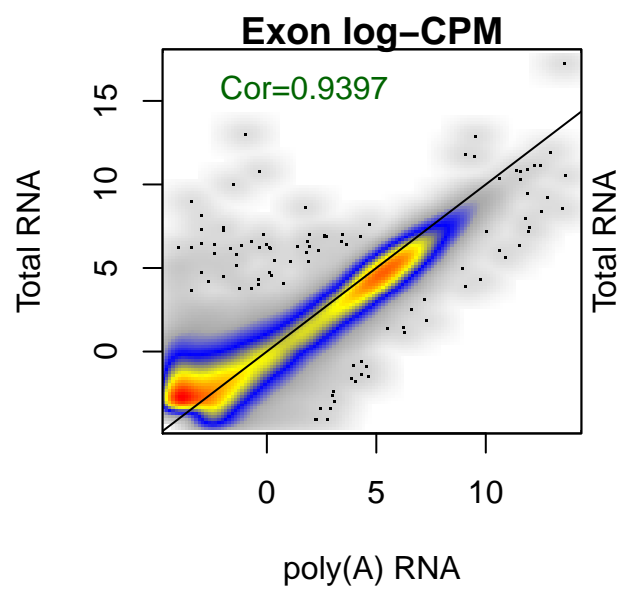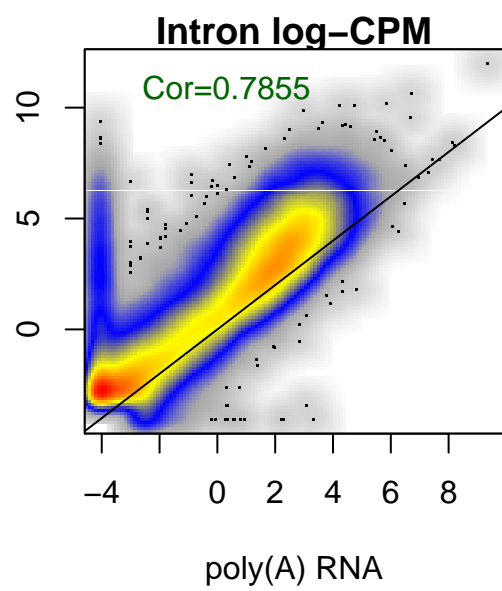

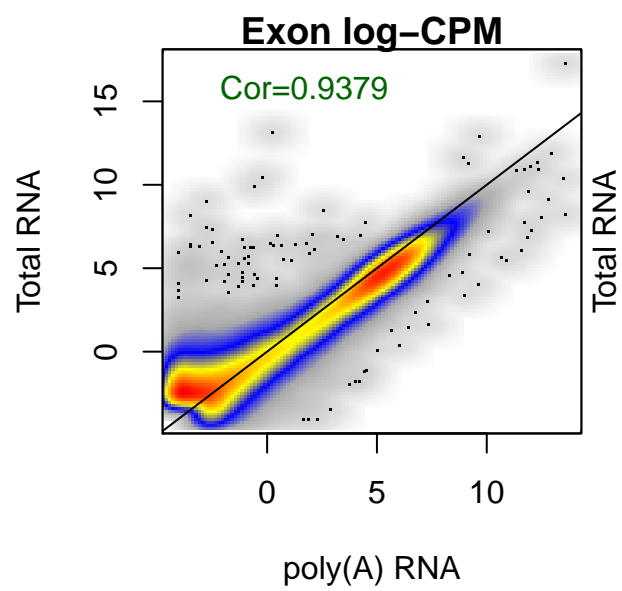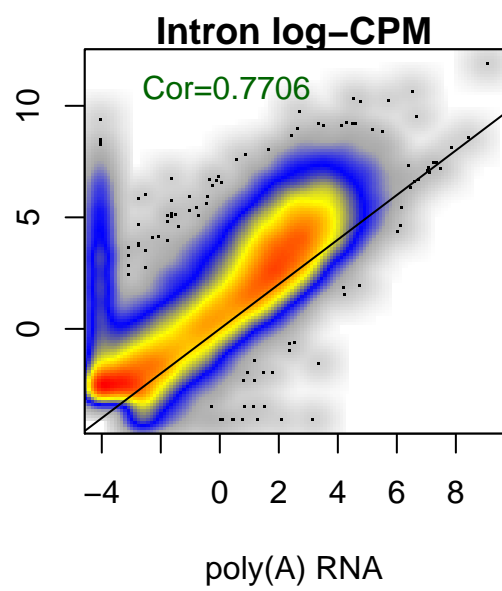

Supplement: lqaa073_Supplemental_Files [file lqaa073_supplemental_files.zip › Sup Fig 1 - Between library comparisons.pdf]

Supplementary Figure S2

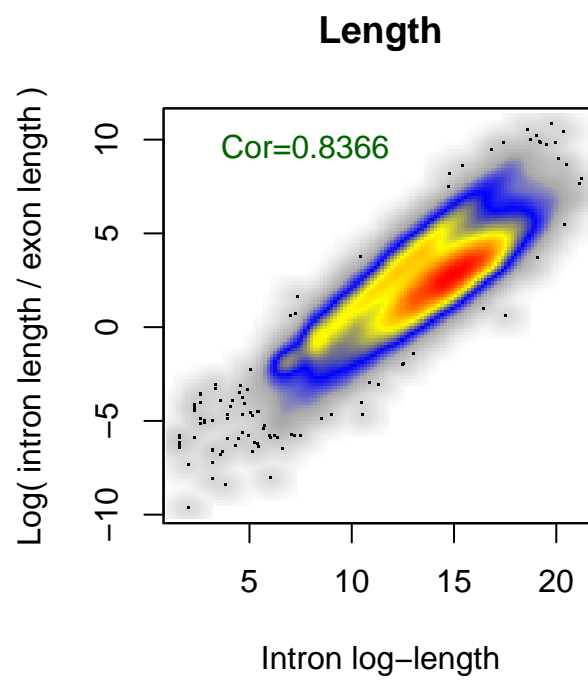

Supplement: lqaa073_Supplemental_Files [file lqaa073_supplemental_files.zip › Sup Fig 2 - Intron length.pdf]

Supplementary Figure S3

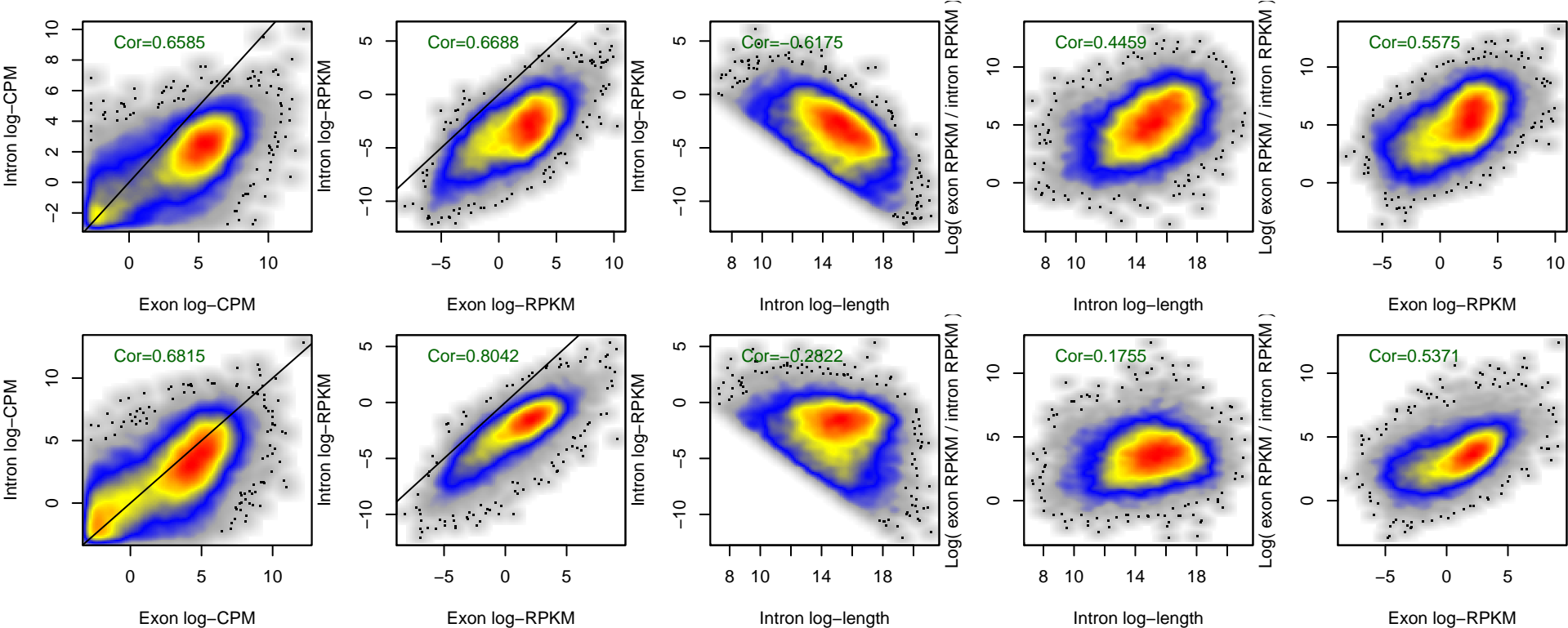

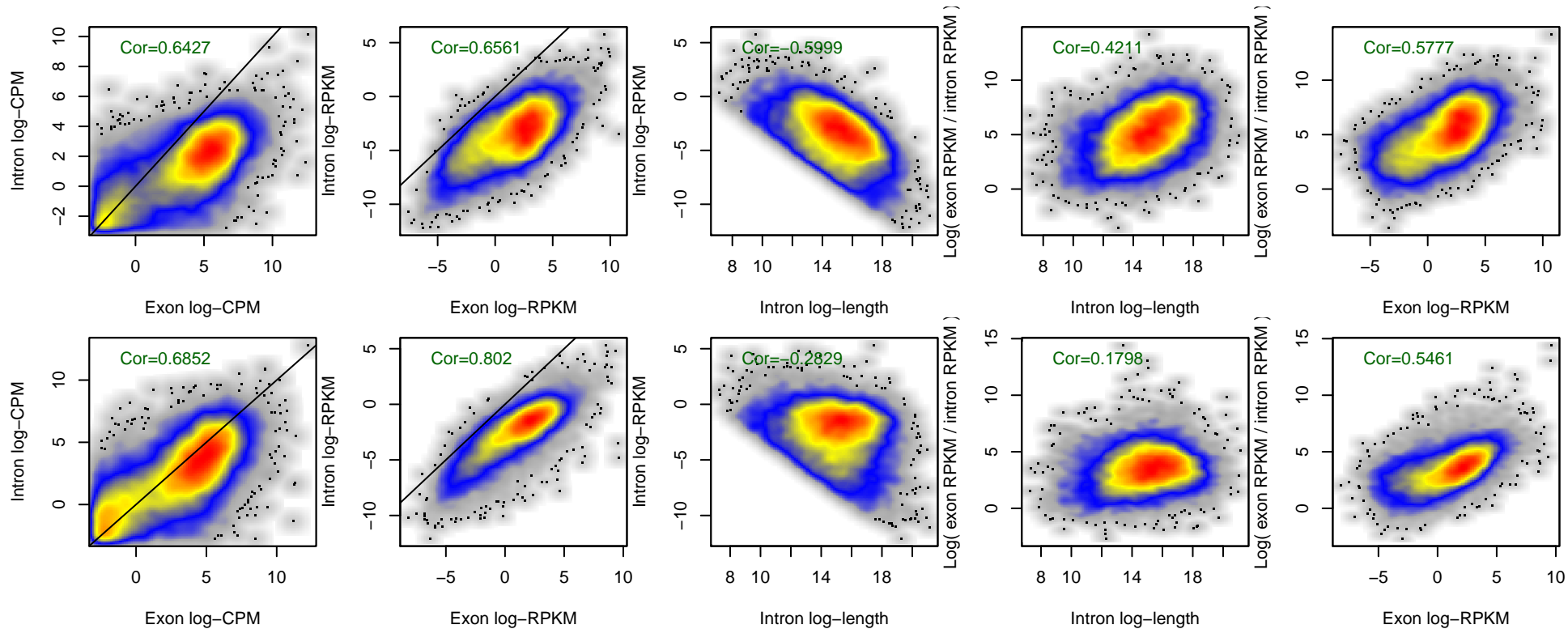

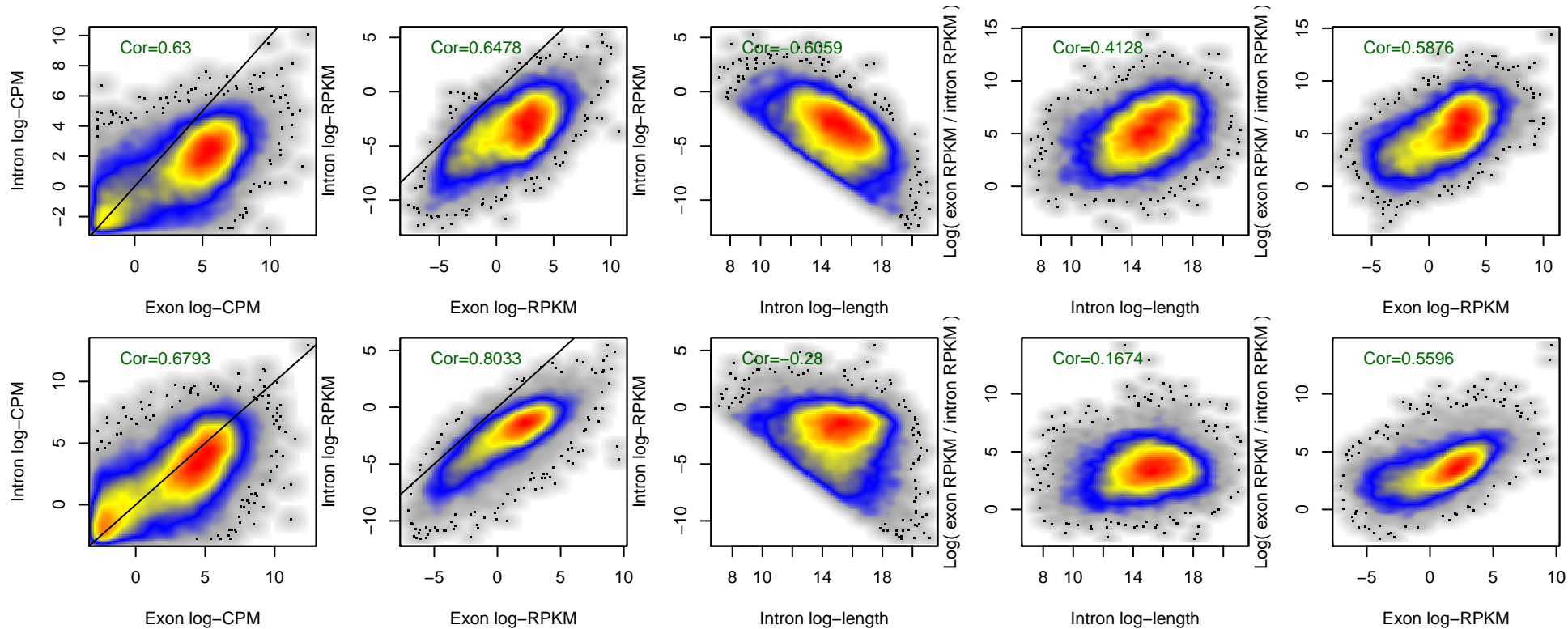

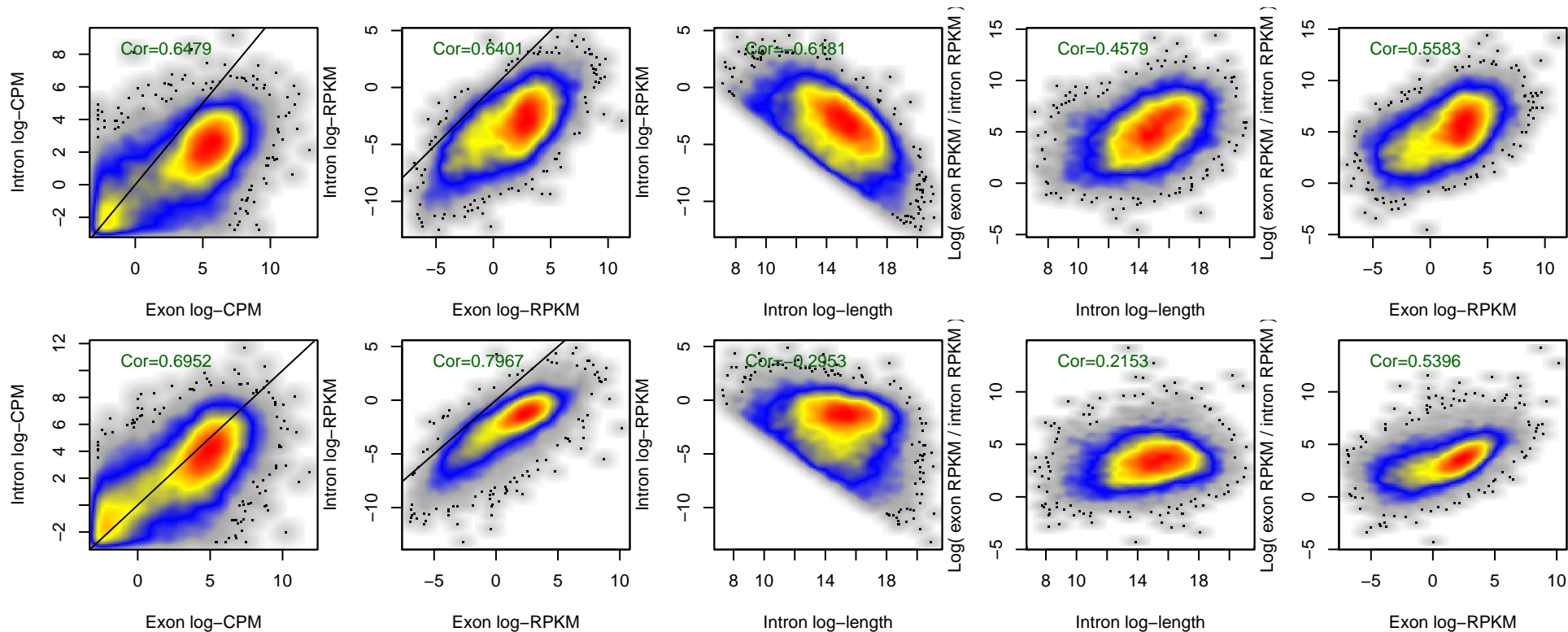

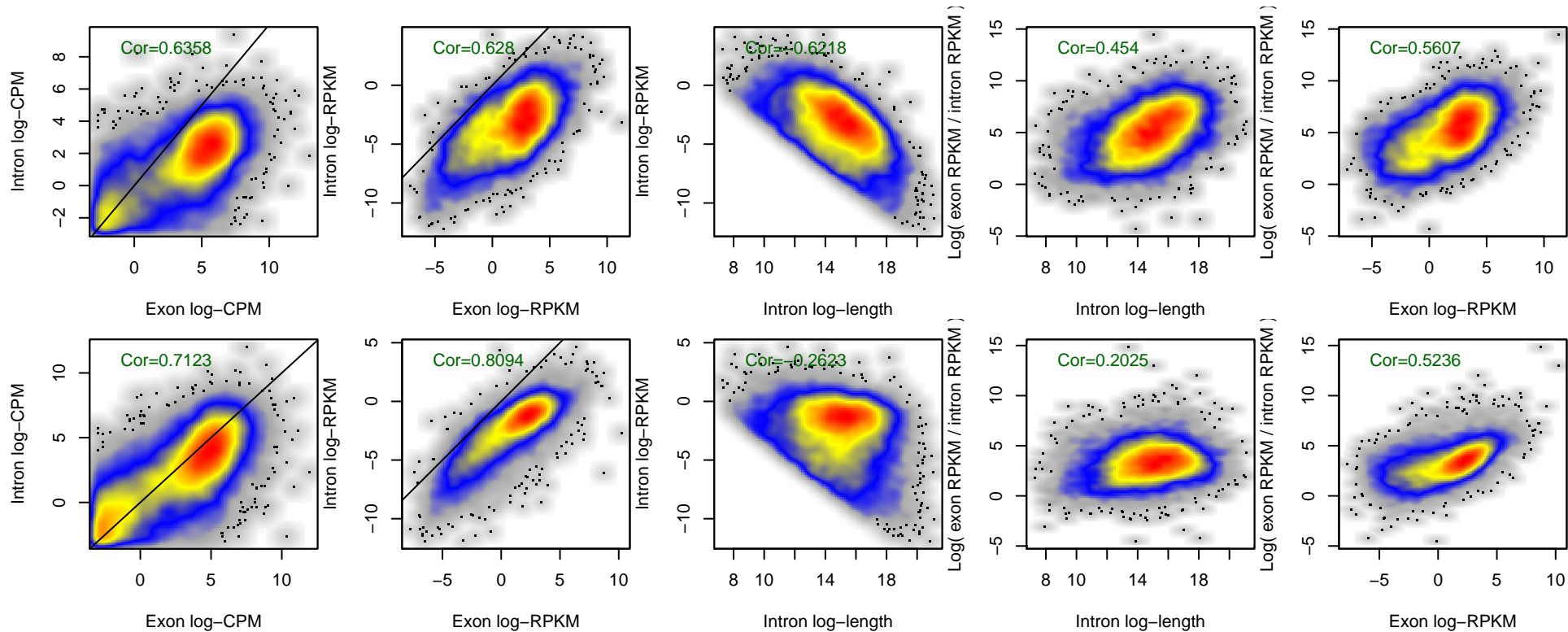

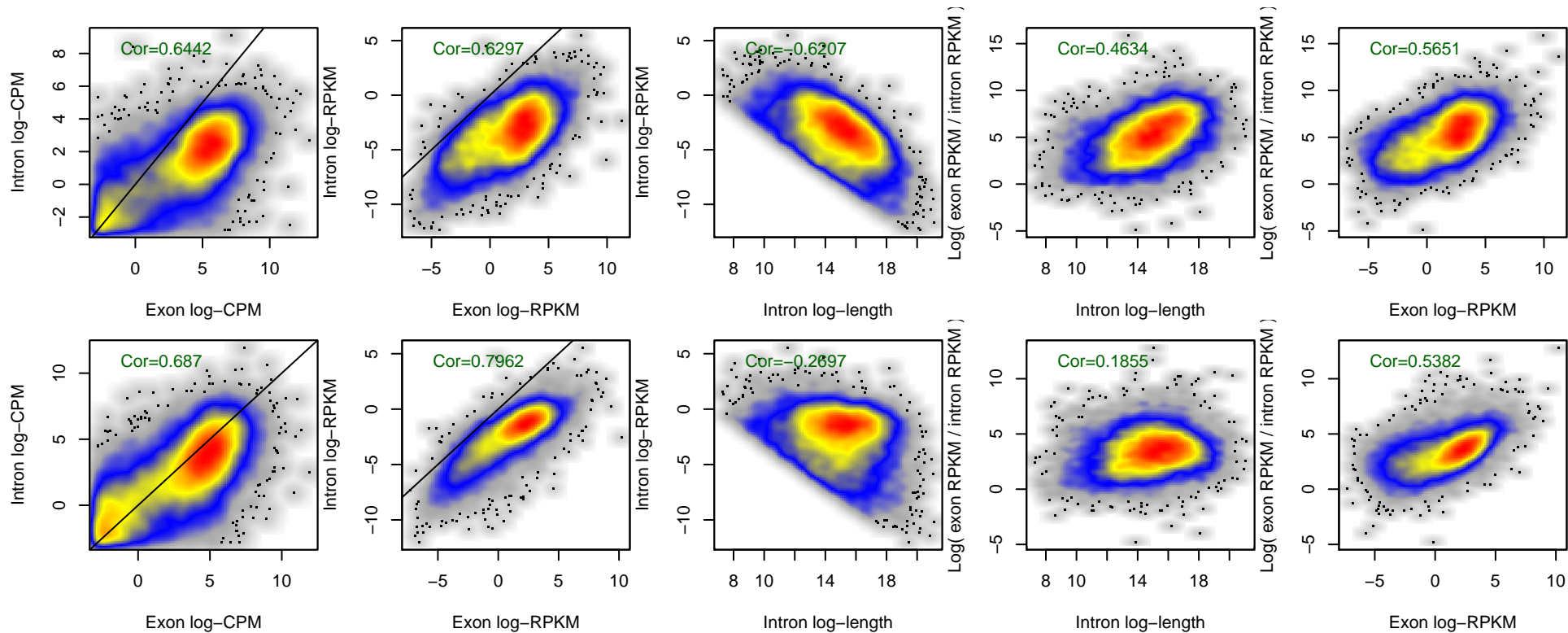

Supplement: lqaa073_Supplemental_Files [file lqaa073_supplemental_files.zip › Sup Fig 3 - Within library comparisons.pdf]

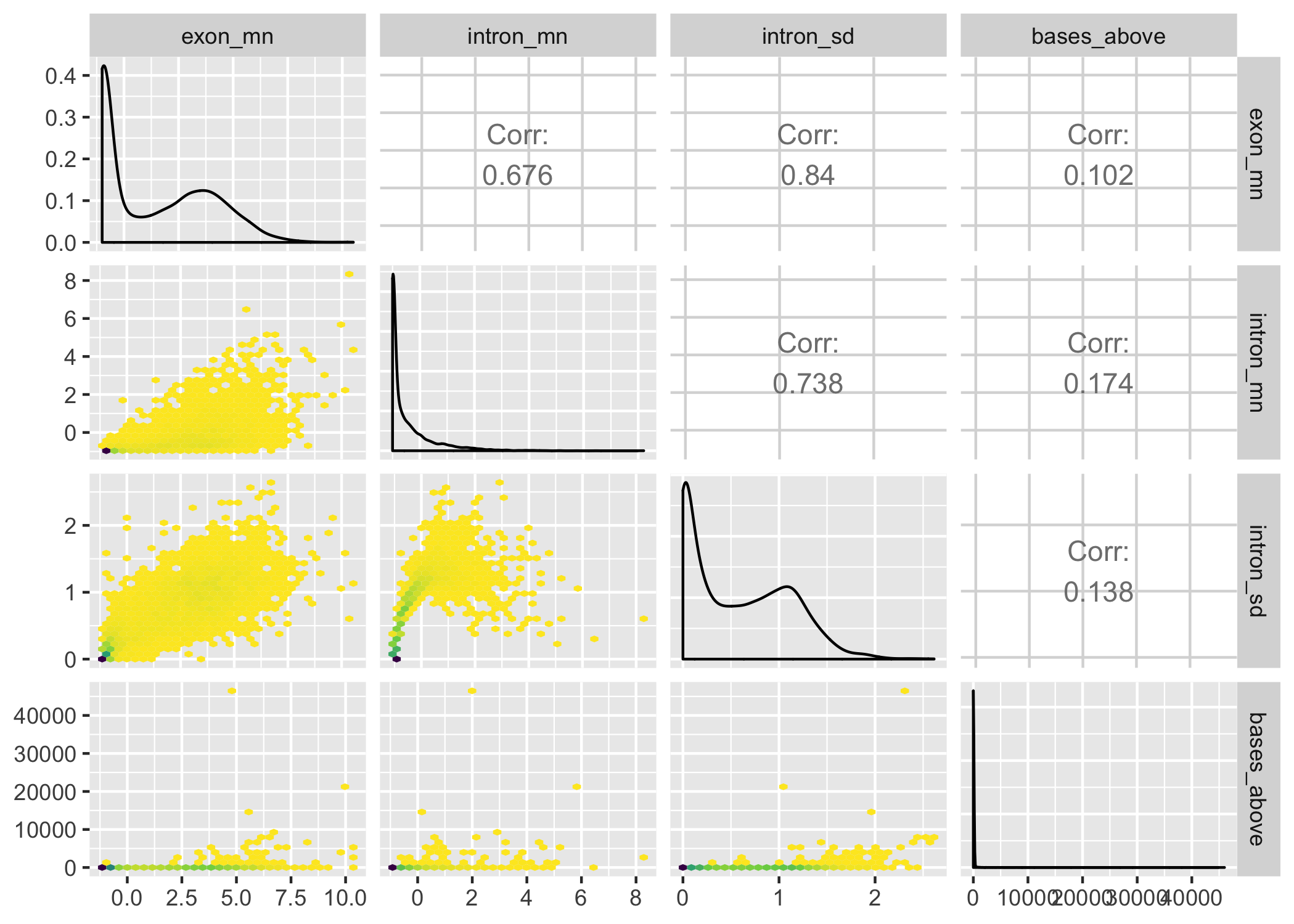

Supplement: lqaa073_Supplemental_Files [file lqaa073_supplemental_files.zip › Sup Fig 4 - Superintronic summary values.png]

(a) Coverage over HNRNPL

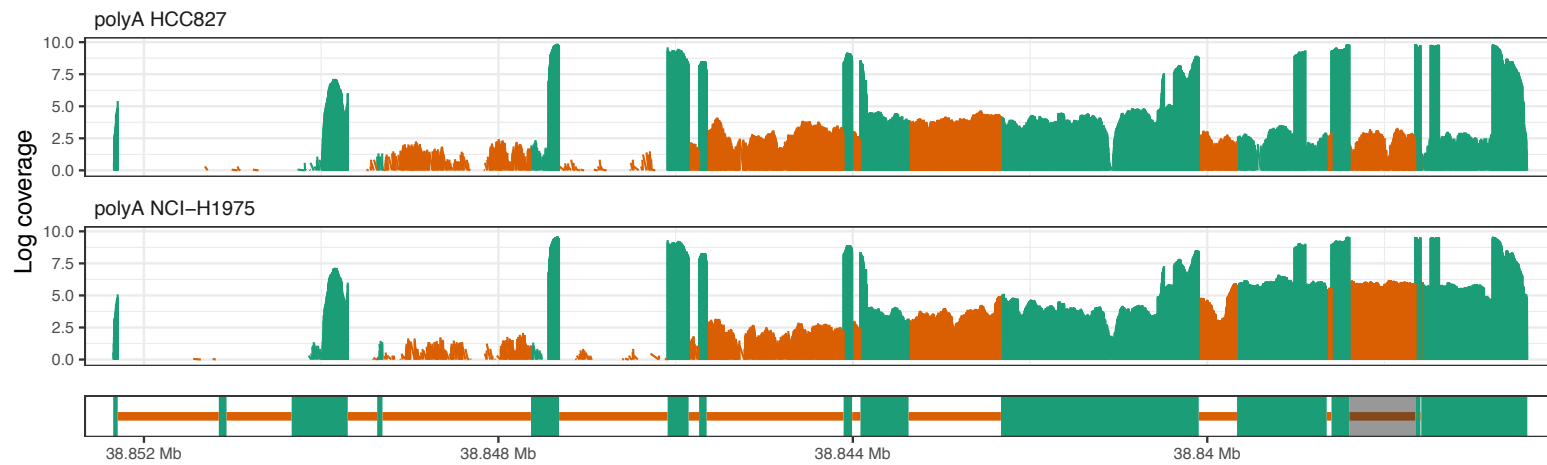

(b) Coverage over NBEAL2

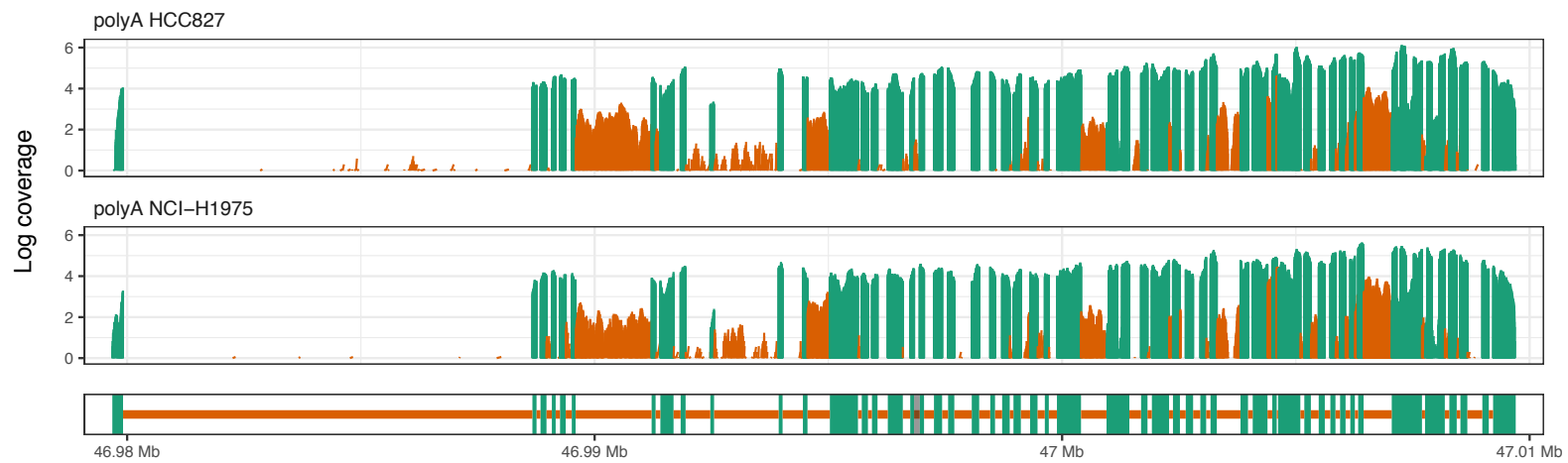

(c) Coverage over HLA-B

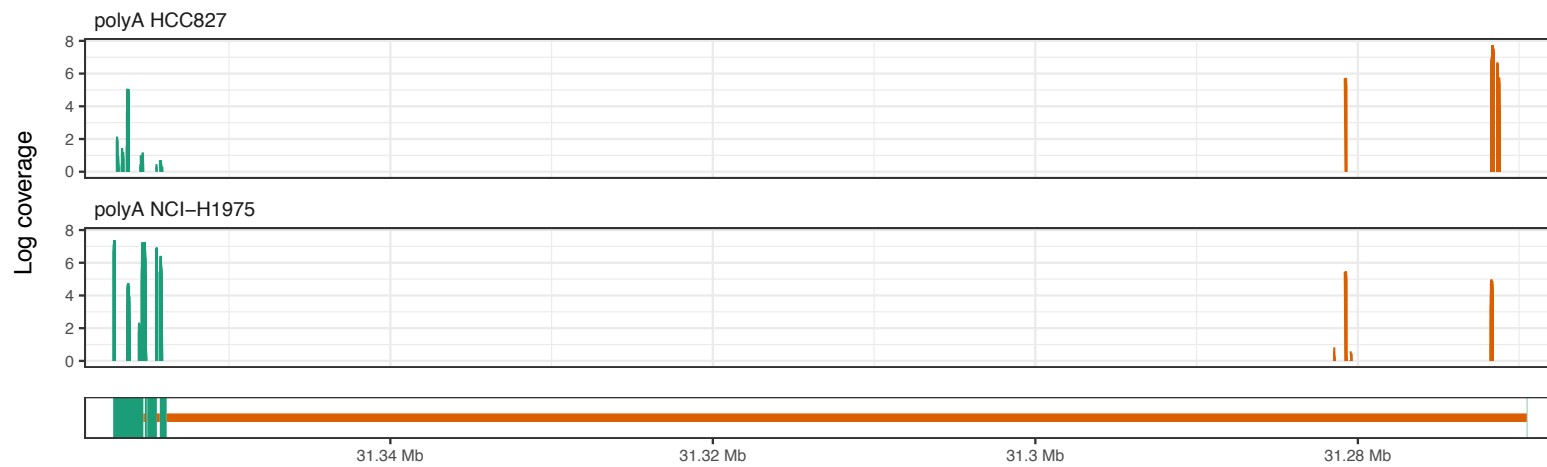

Supplement: lqaa073_Supplemental_Files [file lqaa073_supplemental_files.zip › Sup Fig 5 - Example DIR genes from IRF and ISA.pdf]
